# Cost-effectiveness of two psychological treatments for the reduction of alcohol consumption

**DOI:** 10.1093/eurpub/ckac129.428

**Published:** 2022-10-25

**Authors:** S Flores, I Feldman, F Sampaio

**Affiliations:** Institute for Public Health and Caring Sciences, Uppsala University, Uppsala, Sweden

## Abstract

**Background:**

Up to 7% of the Swedish population meets criteria for harmful use or alcohol dependency but only 10-20% seek treatment. One of the most recommended psychological treatments for controlled drinking is Motivational Enhancement Therapy (MET). Behavioural Self-Control Training (BSCT) is another treatment that is unique in that it is based on the psychology of learning and specifically focused on skills training. To our knowledge, no previous studies exist that evaluated the cost-effectiveness of BSCT for alcohol use disorders (AUD). The aim of this study is to assess the cost-effectiveness of BSCT compared to MET for patients with AUD aiming for controlled drinking over the longer-term from a societal perspective.

**Methods:**

We modelled a cohort of patients with AUD who aim for controlled drinking, over a 10 year time horizon, and estimated the expected costs and outcomes of BSCT and MET. The model reflects the epidemiological transitions between drinking states, which reflect different levels of daily alcohol intake. Each drinking state is connected to temporary or long-term complications attributable to alcohol consumption, different costs and utilities. The data was sourced from a randomized trial evaluating the effectiveness of MET vs BSCT. Risks for complications and associated costs, utilities and mortality were sourced from the literature.

**Results:**

Compared to MET, BSCT resulted in less total QALYs gained (4,7 vs 6,6 QALYs per patient). MET remains a cost effective treatment compared to BSCT [incremental cost-effectiveness ratio (ICER) = SEK 8497,79 per gained QALY] and a No Intervention scenario BSCT [incremental cost-effectiveness ratio (ICER) = SEK 16321,07 per gained QALY] at a threshold of 500.000 SEK per QALY.

**Conclusions:**

This study suggests that MET should remain the recommended treatment for AUD patients with a goal of controlled drinking in favor of BSCT.

**Key messages:**

• Motivational Enhancement Therapy should remain the recommended treatment for AUD patients with controlled drinking as their goal.

• A future study comparing Motivational Enhancemente Therapy to Behaivoral Self Control Training as recommended treatment in patients wanting to achieve abstinenece is suggested.

